# Integrated analysis of HSP20 genes in the developing flesh of peach: identification, expression profiling, and subcellular localization

**DOI:** 10.1186/s12870-023-04621-0

**Published:** 2023-12-21

**Authors:** Chunhua Zhang, Yanping Zhang, Ziwen Su, Zhijun Shen, Hongfeng Song, Zhixiang Cai, Jianlan Xu, Lei Guo, Yuanyuan Zhang, Shaolei Guo, Meng Sun, Shenge Li, Mingliang Yu

**Affiliations:** 1 Institute of Pomology, Jiangsu Academy of Agricultural Sciences/Jiangsu Key Laboratory for Horticultural Crop Genetic Improvement, Nanjing, Jiangsu Province China; 2 Suzhou Polytechnic Institute of Agriculture, Suzhou, Jiangsu Province China

**Keywords:** Peach, HSP20 family, Subfamily classification, Motif composition, Exon/intron organization, Expression profiling, Subcellular location

## Abstract

**Background:**

Plant HSP20s are not only synthesized in response to heat stress but are also involved in plant biotic and abiotic stress resistance, normal metabolism, development, differentiation, survival, ripening, and death. Thus, HSP20 family genes play very important and diverse roles in plants. To our knowledge, HSP20 family genes in peach have not yet been characterized in detail, and little is known about their possible function in the development of red flesh in peach.

**Results:**

In total, 44 PpHSP20 members were identified in the peach genome in this study. Forty-four PpHSP20s were classified into 10 subfamilies, CI, CII, CIII, CV, CVI, CVII, MII, CP, ER, and Po, containing 18, 2, 2, 10, 5, 1, 1, 2, 1, and 2 proteins, respectively. Among the 44 *PpHSP20* genes, 6, 4, 4, 3, 7, 11, 5, and 4 PpHSP20 genes were located on chromosomes 1 to 8, respectively. In particular, approximately 15 *PpHSP20* genes were located at both termini or one terminus of each chromosome. A total of 15 tandem *PpHSP20* genes were found in the peach genome, which belonged to five tandemly duplicated groups. Overall, among the three cultivars, the number of *PpHSP20* genes with higher expression levels in red flesh was greater than that in yellow or white flesh. The expression profiling for most of the *PpHSP20* genes in the red-fleshed ‘BJ’ was higher overall at the S3 stage than at the S2, S4-1, and S4-2 stages, with the S3 stage being a very important period of transformation from a white color to the gradual anthocyanin accumulation in the flesh of this cultivar. The subcellular localizations of 16 out of 19 selected PpHSP20 proteins were in accordance with the corresponding subfamily classification and naming. Additionally, to our knowledge, Prupe.3G034800.1 is the first HSP20 found in plants that has the dual targets of both the endoplasmic reticulum and nucleus.

**Conclusions:**

This study provides a comprehensive understanding of PpHSP20s, lays a foundation for future analyses of the unknown function of *PpHSP20* family genes in red-fleshed peach fruit and advances our understanding of plant *HSP20* genes.

**Supplementary Information:**

The online version contains supplementary material available at 10.1186/s12870-023-04621-0.

## Background

Heat shock proteins (HSPs), also known as molecular chaperones, have been extensively studied since their discovery in the early 1960s by Ferruccio Ritossa [1]. The superfamily of HSPs in eukaryotes is classified into the HSP110, HSP90, HSP70, HSP60, and small heat shock protein (sHSP) families by their approximate molecular weights in kDa [2].

Among these groups, sHSPs are ubiquitous and evolutionally conserved small proteins with molecular weights ranging from 12 to 42 kDa. Their highly conserved sequences (~ 70 amino acids) at the C-terminus are termed α-crystalline domains (ACDs) [3, 4]. The molecular weights of most sHSPs are in the range of 15–22 kDa, and these proteins are therefore also known as HSP20 proteins, low-molecular-weight proteins (LMW proteins), or α-crystallin proteins. The HSP20 family is also known as the LMW family, or α-crystallin family. In addition, less conserved ACDs present in another multidomain protein family referred to as sHSP-like or ACD proteins are also included in the HSP20 family [3].

Plant HSP20s are not only synthesized in response to heat stress [5] but can also be highly induced in plant resistance responses to various biotic and abiotic stresses, such as high salinity, drought, wounding, nematode infection, *Botrytis cinerea*, and tomato spotted wilt virus [6, 7]. Moreover, recent studies in several plants have also revealed that a subset of *HSP20* genes function in normal metabolism, development, differentiation, survival, ripening, and death [4, 8–10]. In angiosperms, based on subcellular localization, amino acid sequence homology, and immunological cross-reactivity, HSP20s are divided into a maximum of 19 subfamilies. There are seven HSP20 subfamilies considered cytosolic/nuclear (CI, CII, CIII, CIV, CV, CVI, and CVII) [11–13]. Recently, new cytosolic/nuclear subfamilies (CVIII, CIX, CX, CXI, and CXII) have also been reported in some plant species [4, 12]. Additionally, there are six subfamilies encoding proteins that are targeted to chloroplasts (CP), mitochondria (MT or M), the endoplasmic reticulum (ER), peroxisomes (Px or Po), or plastids (P) as well as proteins reported to be targeted to both mitochondria and chloroplasts (MTI/CP) [7, 14]. Similar to HSP20 proteins targeted to the cytosol/nucleus, HSP20 proteins targeted to mitochondria are also divided into the MI and MII subfamilies. The maximal number of 19 subfamilies (CI, CII, CIII, CIV, CV, CVI, CVII, CVIII, CIX, CX, CXI, CXII, MI, MII, ER, CP, P, Po/Px, and MTI/CP) represents the most integrated classification of plant HSP20s to date. However, not all HSP20s belong to these 19 conserved subfamilies. Specifically, different plant species have different subfamily numbers among these 19 subfamilies. For instance, in some plants, the subfamilies include P [4, 15], while in other plants, the subfamilies do not include P [13]. The same situation is found for subfamilies CP, CIV, and so on [16].

With the development of sequencing technology, a large number of plant species have been subjected to genome-wide sequencing, making the identification of HSP20 family members achievable. At the genome-wide level, 19, 163, 12, 39, 48, and 42 members of the HSP20 gene family have been reported and analyzed in *Arabidopsis thaliana* (*A. thaliana*) [11], wheat (*Triticum aestivum*) [17], *Malus sieversii* (*M. sieversii*) [18], rice (*Oryza sativa*) [15], grape [13], and tomato (*Solanum lycopersicum*) [7], respectively.

Peach (*Prunus persica*) is a very popular horticultural plant in China. Traditionally, white- and yellow-fleshed fruits have been predominate in the peach market. Recently, red-fleshed peach fruits have also appeared on the market. Red flesh is an attractive and important trait contributing to the commercial and nutritional value of peaches due to its nutritional components benefiting human health. Peach is also the model plant of the Rosaceae family. Its whole-genome sequence was released online in 2013 [19]. Our lab has been seeking to identify the gene controlling the dominant red-fleshed trait for several years. In 2013, 64 predicted genes were found to be located in the red-fleshed trait region (505 kb) [20]. Thereafter, through further fine mapping, the red-fleshed trait region was narrowed to 11 genes. Among these 11 genes, one encodes the HSP20 protein. To date, HSP20 family members associated with development of red flesh in peach have not been reported. Additionally, experimental evidence allowing the assignment of HSP20 paralogs to the above subfamilies has not been provided for peach to our knowledge. Thus, all *HSP20* family genes were identified and characterized, and their subfamily classification, motif composition, gene structure, and expression profiles in red-fleshed fruit (white- and yellow-fleshed fruits were used as controls), and subcellular localizations were analyzed in this study. The findings of this study will provide the necessary foundational knowledge for further functional verification of *HSP20* family genes involved in peach fruit development.

## Results

### Identification of *PpHSP20* family genes

In total, 44 *PpHSP20* genes were yielded from the ‘Lovel’ peach genome under HMM analysis (Table S3). The results from the CD search and InterProScan confirmed that all 44 HSP20 proteins belonged to the HSP20 family. The identities of the *PpHSP20* genes from both the peach v1.0 and v2.0 genomes are shown in Table S3. Among these genes, no corresponding sequence or ID within peach genome v1.0 was found for Prupe.7G008500.1 (Table S3). The lengths of the peptide sequences, CDSs, genomic sequences, and transcript sequences of the 44 PpHSP20 members ranged from 87 to 919 amino acids, 264 to 2760 bp, 365 to 10848 bp, and 264 to 3585 bp, respectively.

The BLAST results from the TAIR database showed that some *PpHSP20* genes had the same *A. thaliana* homologous gene (Table S3); for example, *Prupe.6G066100.1*, *Prupe.6G065900.1*, *Prupe.6G066400.1*, *Prupe.6G066300.1*, and *Prupe.6G066200.1* were all homologous to *AT3G46230.1* (*HSP17.4*) (Table S3 and S1). Similar cases were also found for *Prupe.8G000400.1* and* Prupe.1G586200.1* (homologous to *AT4G10250.1*) and the pair of *Prupe.6G066500.1* and *Prupe.2G243400.1* (homologous to *AT1G07400.1*).

### Phylogenetic relationships and subfamily classification of PpHSP20 proteins

**Fig. 1:**
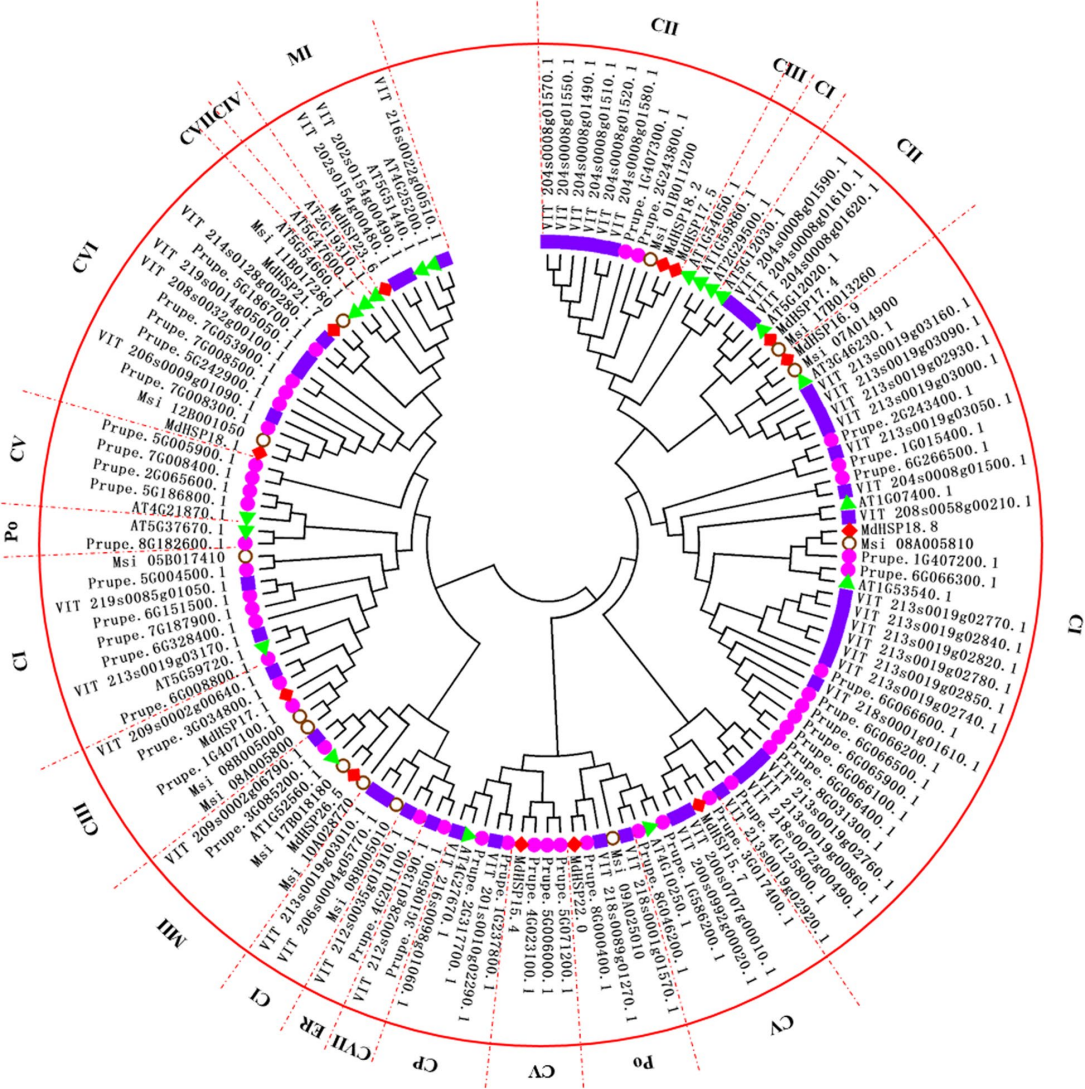
Phylogenetic tree and subfamily classification of the deduced HSP20 proteins from five plant species. The purple circles, green triangles, red rhombi, empty black circles, and purple rectangles represent 44, 19, 13, 13, and 48 HSP20 proteins from peach, *Arabidopsis thaliana*, *Malus domestica* (*M. domestica*), *M. sieversii*, and grape, respectively. The tree was generated with MEGA 4.1 using the neighbor-joining method

Based on the phylogenetic tree (Fig. 1) and references in *Arabidopsis*, apple, grape, and other plant species, the 44 PpHSP20 proteins identified in this study were divided into 10 subfamilies, CI, CII, CIII, CV, CVI, CVII, MII, ER, CP, and Po, containing 18, 2, 2, 10, 5, 1, 1, 1, 2, and 2 members, respectively. For the CIV and MI subfamilies, no PpHSP20 protein was found (Fig. 1).

Prupe.1G407300.1 and Prupe.2G243800.1 were located next to VIT204s0008g01580.1 (VvHSP20-10), VIT204s0008g01520.1 (VvHSP20-7), VIT204s0008g01510.1 (VvHSP20-6), VIT204s0008g01490.1 (VvHSP20-4), VIT204s0008g01550.1 (VvHSP20-8), and VIT204s0008g01570.1 (VvHSP20-9) (Fig. 1; Table S1), which all belong to subfamily CII. Thus, Prupe.1G407300.1 and Prupe.2G243800.1 were classified into subfamily CII in peach. A quarter of the whole circular tree extended from MdHSP16.9, Prupe.2G243400.1 to Prupe.4G125800.1 and included the AT3G46230.1, AT1G07400.1, AT1G53540.1, and 16 VvHSP20 proteins, which mostly belonged to subfamily CI, with two exceptions (VIT204s0008g01500.1 (VvHSP20-5) and VIT218s0001g01610.1 (VvHSP20-46). Thus, PpHSP20 proteins ranging from Prupe.2G243400.1 to Prupe.4G125800.1 were classified into the CI subfamily in peach.

Prupe.3G034800.1 and Prupe.1G407100.1 were on the same branch as VvHSP20-18 (VIT209s0002g00640.1), which belongs to the CIII subfamily in grape [13]. Therefore, Prupe.3G034800.1 and Prupe.1G407100.1 were classified into the CIII subfamily in peach. Prupe.3G017400.1 clustered closely with the VIT200s0707g00010.1 (VvHSP20-47) and VIT200s0992g00020.1 (VvHSP20-48) proteins, which belong to subfamily CV in grape [13]. Thus, Prupe.3G017400.1 was classified into subfamily CV in this study. Prupe.5G071200.1, Prupe.5G006000.1, and Prupe.4G023100.1 were clustered together with MdHSP15.4-CV; therefore, Prupe.5G071200.1, Prupe.5G006000.1, and Prupe.4G023100.1 were classified into subfamily CV in this study. In the tail of the circular tree, Prupe.5G186800.1, Prupe.2G065600.1, Prupe.7G008400.1, and Prupe.5G005900.1 were clustered together with AT4G21870.1, which is AtHSP15.4-CV; therefore, Prupe.5G186800.1, Prupe.2G065600.1, Prupe.7G008400.1, and Prupe.5G005900.1 were classified into subfamily CV in this study.

Both AT4G10250.1 and VIT218s0089g01270.1 (VvHSP20-42) belong to the ER subfamily, and VIT218s0001g01570.1 (VvHSP20-45) belongs to the Po or Px subfamily. Prupe.1G586200.1, Prupe.8G046200.1, Prupe.8G000400.1, AT4G10250.1, VvHSP20-42, and VvHSP20-45 were all found to be on the same branch in this study (Fig. 1). Thus, Prupe.1G586200.1, Prupe.8G046200.1, and Prupe.8G000400.1 were originally classified into the ER subfamily. However, based on recent experimental evidence of subcellular location, Prupe.1G586200.1 and Prupe.8G046200.1 were finally classified into neighboring subfamily CV. Prupe.8G000400.1 was classified into subfamily Po based on experimental evidence of subcellular location.

Prupe.4G201100.1 and VIT212s0028g01390.1 (VvHSP20-21, an ER subfamily protein) clustered together. Thus, Prupe.4G201100.1 was classified into the ER subfamily. Prupe.3G085200.1 was located between two MII homologous proteins, VIT209s0002g06790.1 (VvHSP20-19) and AT1G52560, so Prupe.3G085200.1 was classified into subfamily MII in peach. Prupe.1G237800.1 and Prupe.2G317700.1, together with three subfamily CP members, VIT201s0010g02290.1 (VvHSP20-1), VIT216s0098g01060.1 (VvHSP20-40), and AT4G27670 (AtHsp25.3), were located on the same branch. Thus, Prupe.1G237800.1 and Prupe.2G317700.1 were classified into subfamily CP.

### Conserved motif and physiochemical characteristics of PpHSP20 proteins

**Fig. 2:**
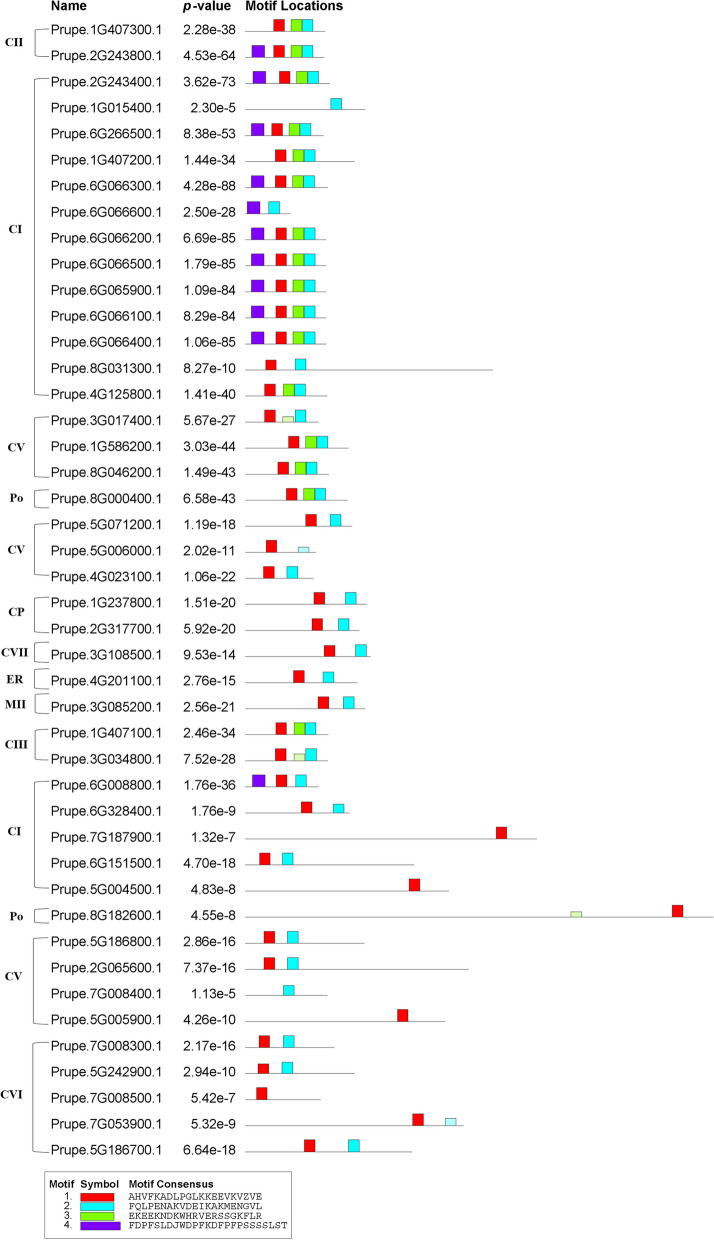
Locations of conserved motifs in PpHSP20 family proteins. The IDs of PpHSP20s is arranged in order of the Fig. 1. The red, blue, green, and purple boxes on the gray lines are four kinds of conserved motifs in the deduced protein sequences of PpHSP20 proteins

**Fig. 3:**
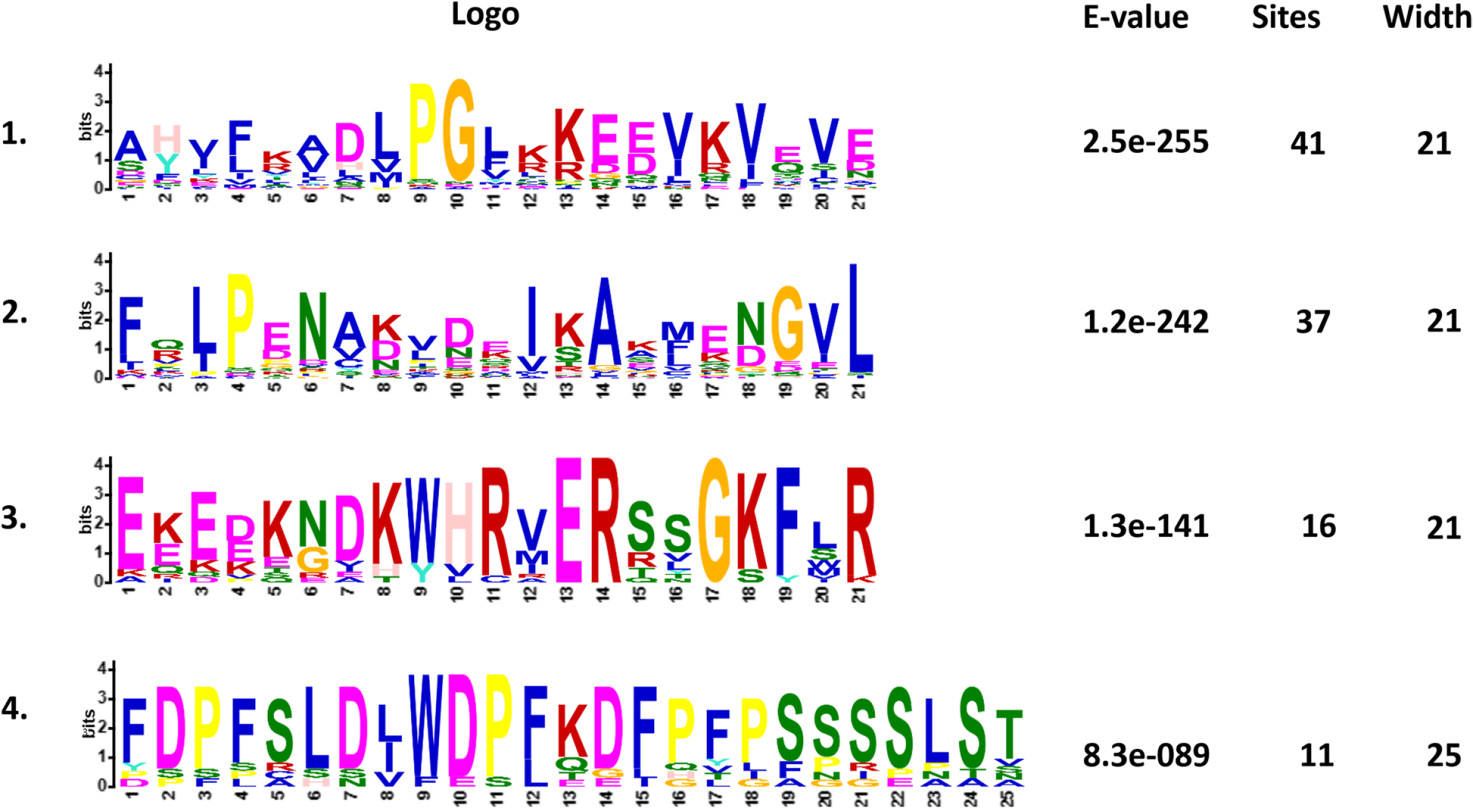
Detailed information of conserved motifs owned by deduced PpHSP20 family proteins in peach. The numbers on the left side represent 4 different motifs, which correspond to motif 1 to motif 4 in Fig. 2. The letters with different sizes and colors imply the types of amino acid residues and predicted reliability in the logo of each conserved motif

Most members of subfamilies CI and CII in peach had four conserved motifs (motifs 1, 2, 3, and 4) (Fig. 2), while most members of subfamilies MII, CP, ER, CV, and CVII had two conserved motifs (motifs 1 and 2). The reliability and logo sequence of each motif are shown in Fig. 3. The reliability order for PpHSP20 was as follows: motifs 1, 2, 3, and 4. Motif 1 (red), with the highest E-value (2.5e^−255^), was the most conserved of the four motifs and appeared in 42 out of 44 PpHSP20 proteins. Prupe.7G008400.1 and Prupe.1G015400.1 do not contain motif 1 in Fig. 2, but according to the NCBI CD search website, they contain a conserved ACD domain and belong to the HSP20 family. Motif 2 (blue), with the second highest E-value (1.2e^−242^), ranked second among the four conserved motifs and appeared in 37 out of 44 PpHSP20 proteins. Most members of subfamilies CI and CII also showed conserved motif orders and conserved clearance between motifs, where from the start to the end of each protein, the motif order was 4, 1, 3, and 2. For the remaining members of the PpHSP20 family, the motif order was mostly 1, 2.

The physiochemical characteristics of the PpHSP20 proteins were predicted to obtain more detailed information about the PpHSP20 family (Table S4). The sizes of 44 PpHSP20 proteins were from 9.40879 kDa (Prupe.6G066600.1) to 100.08987 kDa (Prupe.8G182600.1). The sizes of most of the 44 PpHSP20 proteins were from 14.81327 kDa (Prupe.5G006000.1) to 37.39003 kDa (Prupe.5G186700.1). Sizes exceeding 43 kDa were found for five PpHSP20 proteins, including Prupe.5G004500.1, Prupe.2G065600.1, Prupe.7G053900.1, Prupe.8G031300.1, and Prupe.7G187900.1, and were 44.14085,47.39943, 48.04114, 54.40011, and 62.9442 kDa, respectively.

### Exon/intron structures and distribution on chromosomes of *PpHSP20* genes

**Fig. 4:**
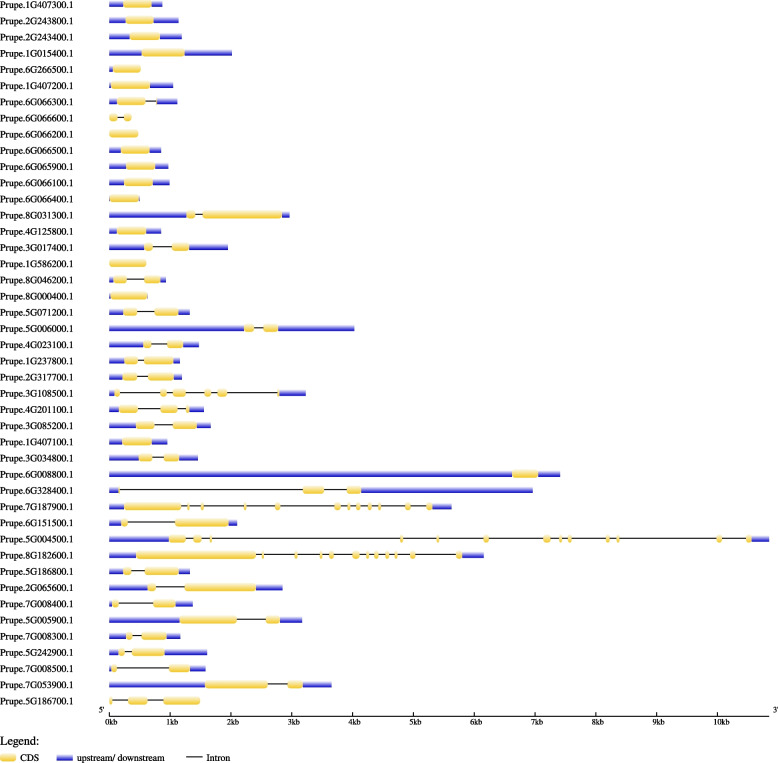
Exon/intron distribution of *PpHSP20* family genes. The distribution and number of exons/introns were analyzed by the online tool GSDS. The ID’s order is in accord with that in Fig. 1. The yellow and blue bars indicate the exons and regions of the 3’UTR and 5’UTR, respectively. The black lines between or among yellow bars indicate the introns. The black line and numbers at the bottom indicate the length of each *PpHSP20* genome sequence

Almost all of the *PpHSP20* genes in subfamilies CP, CV, and CVI included two exons and one intron, except for Prupe.5G186700.1, with three exons and two introns, and Prupe.1G586200.1, with only one exon (Fig. 4). Most of the *PpHSP20* genes in subfamily CI (Fig. 4) and CII owned one exon and no introns. However, in the second subset of subfamily CI, two members (Prupe.7G187900.1 and Prupe.5G004500.1) contained multiple exons and introns (12 exons/11 introns and 13 exons/12 introns, respectively). One member (Prupe.8G182600.1) in subfamily Po also had 12 exons and 11 introns.

**Fig. 5:**
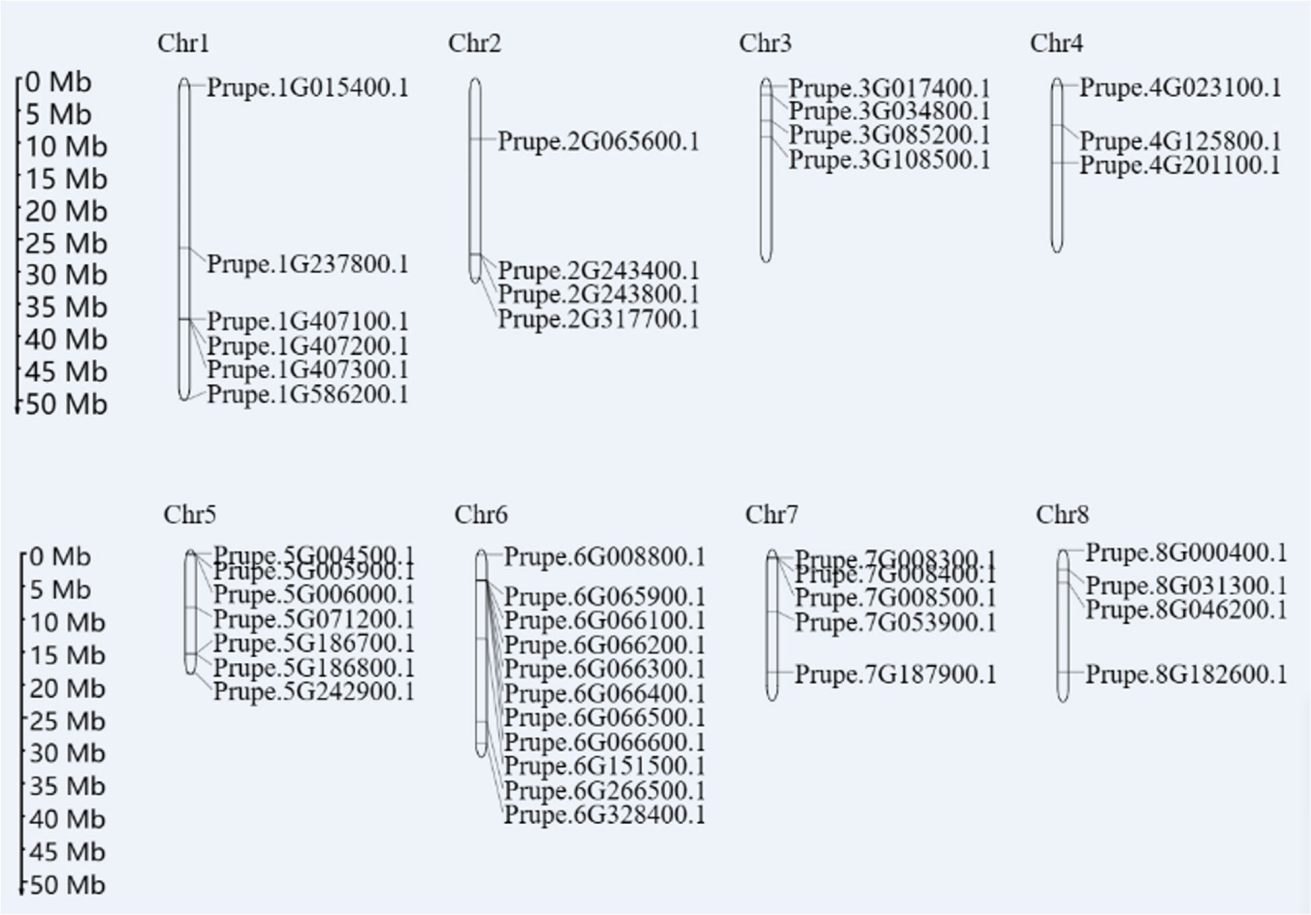
Localization of *PpHSP20* family genes on each chromosome in peach. The distribution of *PpHSP20* genes on the eight chromosomes was visualized using the software MapDrawV2.1

Based on the position on the chromosome of each *PpHSP20* gene (S3 Table) and the length of each chromosome obtained from the GDR, the chromosomal location of each *PpHSP20* gene was mapped to each chromosome using software to intuitively display their positions (Fig. 5). The 44 *PpHSP20* genes were unevenly distributed across chromosomes 1 to 8 of the peach genome. The numbers of *PpHSP20* genes distributed on chromosomes 1 to 8 were 6, 4, 4, 3, 7, 11, 5, and 4, respectively (Table S3 and Fig. 5). Chromosomes 6 and 4 in peach had the highest number (11) and the lowest number (3) of *PpHSP20* genes, respectively.

To investigate the expansion and evolution of the PpHSP20 gene family, gene duplication events in the peach genome were studied. On chromosome 6 (Fig. 5), seven PpHSP20 genes (Prupe.6G065900.1, Prupe.6G066600.1, Prupe.6G066100.1, Prupe.6G066200.1, Prupe.6G066300.1, Prupe.6G066400.1, Prupe.6G066500.1, Prupe.6G066600.1) were positioned next to each other in tandem. The same situation was also found on chromosome 1 (Prupe.1G407100.1, Prupe.1G407200.1, Prupe.1G407300.1) and on chromosome 7 (Prupe.7G008300.1, Prupe.7G008400.1, Prupe.7G008500.1). Two pairs of PpHSP20 genes (Prupe.5G186800.1 and Prupe.5G186700.1, Prupe.5G005900.1 and Prupe.5G006000.1) were also located next to each other on chromosome 5. These tandem genes revealed that tandem duplications might contribute to the evolution and amplification of PpHSP20 family genes in peach.

### Collinearity analysis of *PpHSP20* genes

**Fig. 6:**
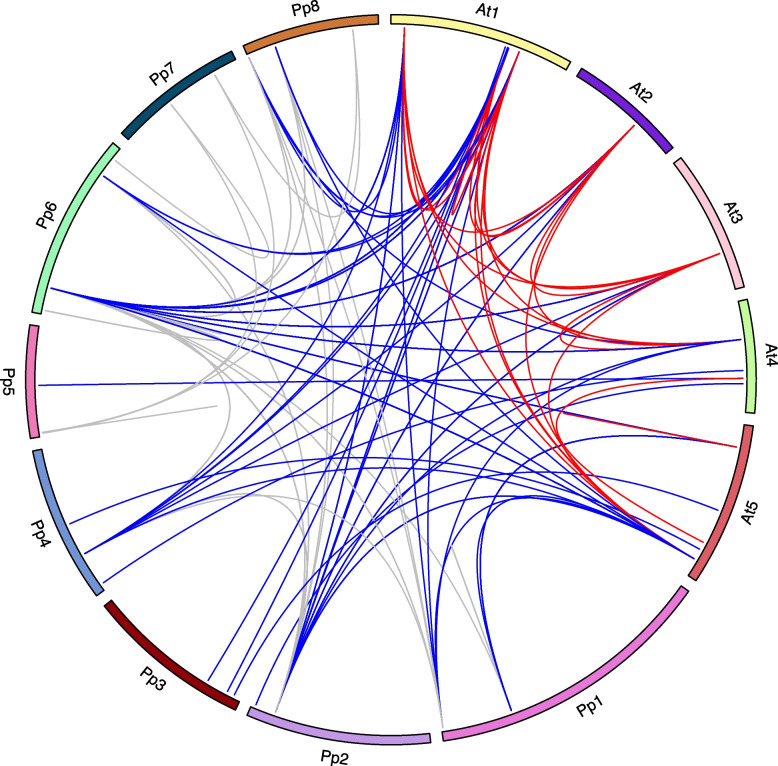
Collinearity analysis of HSP20 family genes in peach and *Arabidopsis thaliana*. The gray lines indicate collinear *HSP20* gene pairs within the peach genome, while the blue lines show the collinear *HSP20* gene pairs between the peach genome and *Arabidopsis thaliana* genome. The red lines show collinear *HSP20* gene pairs within the *Arabidopsis thaliana* genome. The collinearity map was generated by the circlize tool in the R package

In order to further predict the origin, evolutionary relationship and function of the *PpHSP20* family genes, a comparative collinearity map of *HSP20* gene pairs in the peach and *Arabidopsis* genomes was created (Fig. 6). The results revealed that 79, 94, and 21 pairs of *HSP20* collinear genes were identified in peach, peach and *Arabidopsis*, *Arabidopsis*, respectively (Fig. 6 and Table S5).

### Expression profiling of *PpHSP20* family genes

**Fig. 7:**
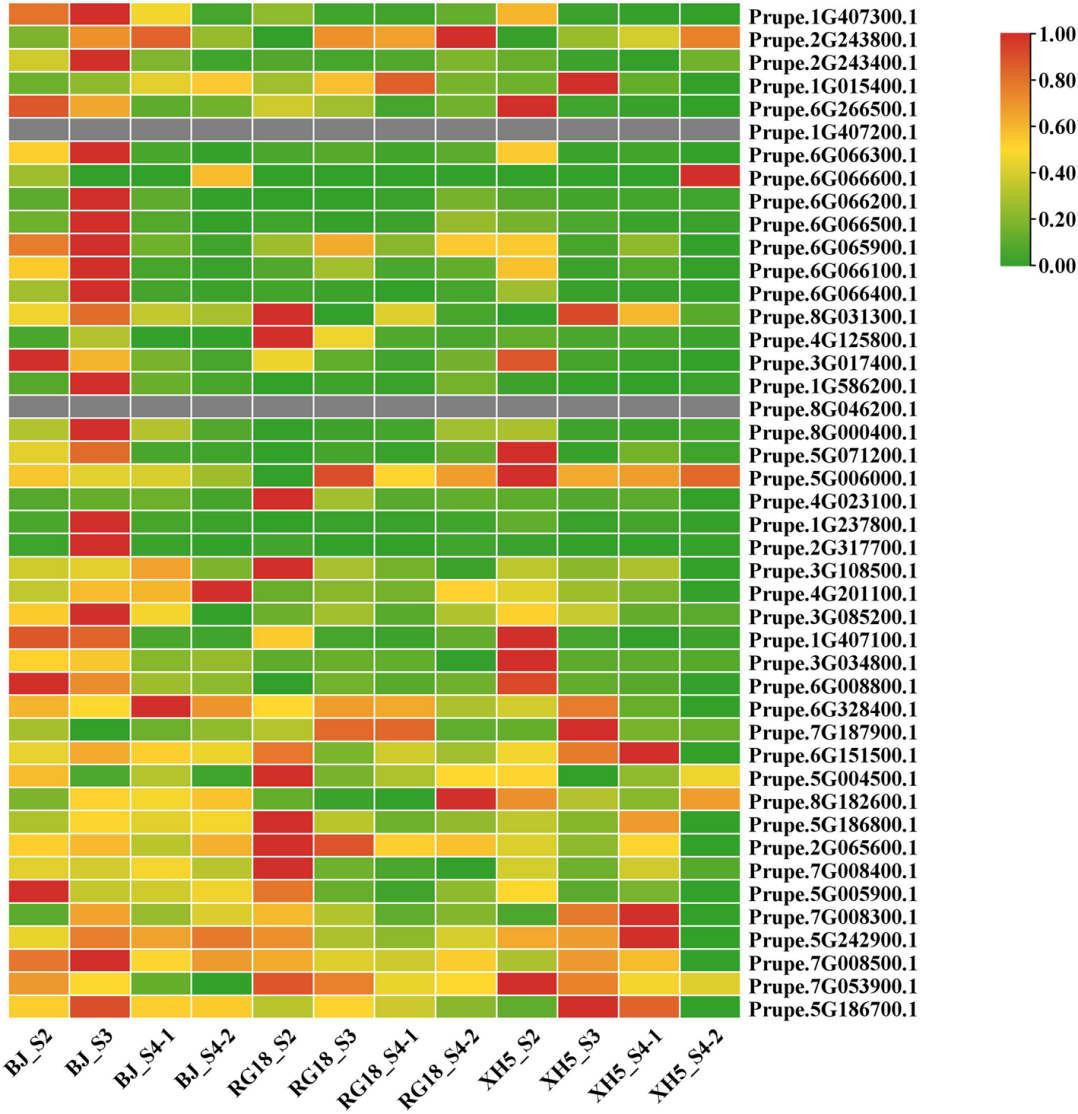
Expression profiling of 44 *PpHSP20* genes at four developmental stages in three cultivars. The cultivars are abbreviated ‘BJ’, ‘XH5’, and ‘RG18’. The four developmental stages were as follows: S2, pit hardening stage; S3, expansion stage; S4-1, premature stage; and S4-2, mature stage. The average FPKM values of three biological repetitions of each developmental stage in each cultivar for each *PpHSP20* gene were extracted from the transcriptomic data. The expression profiles of the 44 *PpHSP20* family genes were generated by the Pheatmap in the R package

To pick out PpHSP20 genes that have different expression patterns between red-fleshed fruit and non-red-fleshed fruit (white- and yellow-fleshed fruits were used as controls), we analyzed the expression profiles of 44 inclined genes in the white-fleshed fruit of ‘XH5’, yellow-fleshed ‘RG18’, and red-fleshed fruit of ‘BJ’ sampled at four developmental stages (Fig. 7). At the same time, inclined genes, which have similar expression patterns between red-fleshed and non-red-fleshed fruits, were also picked out and will not be further investigated by us in the future. In the comparison among red-, yellow-, and white-fleshed fruits at 12 points, *Prupe.1G407300.1* exhibited a higher expression level in the red-fleshed ‘BJ’ than in the white-fleshed fruit of ‘XH5’ and yellow-fleshed fruit of ‘RG18’. Additionally, *Prupe.1G407300.1* and *Prupe.6G266500.1* were characterized by higher expression at stages S2 and S3 than at S4-1 and S4-2 in the red-fleshed ‘BJ’. Overall, as flesh developed from green to mature coloration, the expression levels of *Prupe.1G407300.1* and *Prupe.6G266500.1* decreased gradually in the red-fleshed ‘BJ’, white-fleshed ‘XH5’, and yellow-fleshed ‘RG18’. In contrast, *Prupe.2G243800.1* exhibited lower expression levels at stage S2 than at S3, S4-1, and S4-2 in white-fleshed ‘XH5’ and yellow-fleshed ‘RG18’. Specifically, as flesh developed from green to mature coloration, the expression level of *Prupe.2G243800.1* increased gradually in white-fleshed ‘XH5’ and yellow-fleshed ‘RG18’. The lower expression level of *Prupe.2G243800.1* was investigated at S3 and S4-1 in comparison with that at S2 and S4-2 in the red-fleshed ‘BJ’. Specifically, the expression level of *Prupe.2G243800.1* at the green and mature stages was lower than that in the swelling period and before maturation in the red-fleshed ‘BJ’.

The expression level of *Prupe.1G015400.1* increased gradually as flesh development progressed from the young to mature stages in the red-fleshed ‘BJ’. However, *Prupe.1G015400.1* was highly expressed at S3 and S4-1 but expressed at low levels at S2 and S4-2 in white-fleshed ‘XH5’ and yellow-fleshed ‘RG18’.

*Prupe.1G407200.1* and *Prupe.8G046200.1* showed no expression in these three cultivars at any developmental stage.

The expression level of *Prupe.7G053900.1* showed the same gradual decreasing trend in all three cultivars as the flesh developed from the young green small to mature stages. However, the transcript level of *Prupe.7G053900.1* at each stage in ‘BJ’ was lower than that in both two non–red-fleshed culitvars. *Prupe.1G237800.1* and *Prupe.2G317700.1*, which belong to subfamily CP members, only showed very high levels of transcript in the S3 of red-fleshed ‘BJ’. However, the profiles of *Prupe.1G237800.1* and *Prupe.2G317700.1* at the other three developmental stages of red-fleshed ‘BJ’ and the four stages of non-red-fleshed cultivars were almost the same and were lower than those at S3 in the red-fleshed ‘BJ’.

*Prupe.5G186700.1* accumulated higher levels of transcript at S3 and S4-1 but lower levels at S2 and S4-2 in the two non-red-fleshed cultivars. Higher levels of *Prupe.5G186700.1* transcript at S3 were detected in red-fleshed ‘BJ’ compared with that at the three other stages, which exhibited the same levels at stages S2, S4-1, and S4-2. In particular, we found that the *Prupe.5G186700.1* transcript expressed at S2 and S4-2 in the red-fleshed ‘BJ’ was higher than that in the two cultivars with non-red flesh.

### Subcellular localization of PpHSP20 family proteins

The ORF lengths of 19 *PpHSP20* genes are shown in Table S2. The subcellular localizations of 19 selected PpHSP20 proteins from subfamilies CI, CII, CIII, CV, CVI, MII, Po, and CP were analyzed to verify the subfamily classification of PpHSP20s, which was based on the phylogenetic relationships of peach, grape, *A. thaliana*, *M. domestica*, and *M. sieversii* in this study (Fig. 1) as well as the subfamily classification of apple, grape and *A. thaliana* in previous studies [11, 13].

Six (Prupe.2G243400.1, Prupe.6G066500.1, Prupe.6G065900.1, Prupe.6G066100.1, Prupe.4G125800.1, Prupe.6G008800.1) out of eight analyzed PpHSP20 proteins of subfamily CI had the same subcellular localizations, in the plasma membrane, cytoplasm and nucleus. Two (Prupe.1G407200.1 and Prupe.6G066300.1) out of eight PpHSP20 proteins of subfamily CI (Fig. S1) had slightly different subcellular localizations compared with the above six proteins, as they were located in the cytoplasmic and plasma membrane compartments but not in the nucleus.

Prupe.1G407300.1 of subfamily CII was targeted to the plasma membrane and cytoplasm, and the other detected Prupe.2G243800.1 of subfamily CII was targeted to the plasma membrane, nucleus, and cytoplasm. Prupe.3G034800.1 in subfamily CIII (Fig. 1) was found to be located in the nucleus and endoplasmic reticulum in this study (Fig. S1).

The targeting of Prupe.3G017400.1 in subfamily CV was to the plasma membrane, nucleus, and cytoplasm. Prupe.5G071200.1 of subfamily CV was localized in the plasma membrane, along with intermittent bright foci in the cytoplasm and plasma membrane (Fig. S1). Prupe.7G053900.1 from the subfamily CVI was also localized in the plasma membrane, nucleus, and cytoplasm. Additionally, the intensity of GFP in the nucleus of Prupe.7G053900.1 was much higher than that in the plasma membrane. For Prupe.1G586200.1 and Prupe.8G046200.1 in subfamily CV, the former was localized in the plasma membrane and cytoplasm, and the latter was localized in the plasma membrane, nucleus, and cytoplasm.

Prupe.1G237800.1 in subfamily CP was a chloroplast-localized protein. The localization of Prupe.3G085200.1 of the subfamily MII was the plasma membrane and mitochondria. The subcellular localization of Prupe.8G000400.1 in subfamily Po included the plasma membrane, cytoplasm, and peroxisome.

## Discussion

### Identification and characteristics of PpHSP20 family members

In this study, 44 HSP20 family genes were identified in peach, similar to the number in other plant species. For example, 12, 19, 30, 35, 39, 42, 44, 48, 48, 51, and 163 members of the HSP20 gene family were reported in *M. sieversii* [18], *A. thaliana* [11], cucumber (*Cucumis sativus*) [21], pepper (*Capsicum annuum*) [22], rice (*Oryza sativa*) [15], tomato (*Solanum lycopersicum*) [7], watermelon (*Citrullus lanatus*) [23], grape [13], potato (*Solanum tuberosum*) [16], soybean (*Glycine max*) [12], and wheat (*Triticum aestivum*) [17], respectively. Based on these results, it can be concluded that the PpHSP20 gene family is relatively small. The PpHSP20 gene family is not as large as the MYB and WD40 superfamilies, which have more than two hundred members each [24, 25].

In this study, the number of subfamilies in the PpHSP20 family (10 subfamilies) (Fig. 1) was less than that in cucumber (11 subfamilies) [21], grape (11 subfamilies) [13], rice (14 subfamilies) [4], *A. thaliana* [11] (12 subfamilies), soybean (15 subfamilies) [12], maize (15 subfamilies) [26], and tomato (17 subfamilies) [7]. This confirmed previous findings indicating that HSP20s can be divided into different subfamilies based on cellular localization and sequence homology [26], and not all HSP20s had corresponding homologous proteins in each subfamily of the 12 subfamilies of *A. thaliana*. In particular, some HSP20s are not members of any of the subfamilies of a given plant and are currently considered ‘orphan’ HSP20s [15, 16, 27], which have not been verified by subcellular localization experiments. In plants, the HSP20 family contains more diverse subfamilies than other HSP families with respect to sequence similarity and cellular localization [28]. However, seven subfamilies of plant HSP20s (CI, CII, CIII, ER, CP, M, and Po) identified to date are relatively more conserved among plant species [11].

No PpHSP20 protein was found in the CIV and MI subfamilies in this study (Fig. 1). Similar situations are found in other plant species. For instance, no *SlHSP20* genes have been classified into four subfamilies (CIV, CVIII, CX, and MII subfamilies) in tomato. No *VvHSP20* genes from the grape genome have been classified into five subfamilies, CIV, CVIII, CIX, CX, and CXI. In pepper, the HSP20 CIV, CV, CVIII, CIX, CX, and CXI subfamilies have been found to be absent. In addition, the HSP20 family of rice lacks members of subfamilies CIV and CVII [4, 15]. Several subfamilies, including CIII, CX, CXI, CIX, and CVII, have not been identified among the *CsHSP20* genes of cucumber. Notably, both cucumber and *A. thaliana* have *HSP20* genes in the subfamily CIV, which is absent in peach, grape, pepper, rice, potato, and so on. These results illustrate that gene acquisition and loss events might have occurred during evolution according to the specific developmental, growth, and stress resistance needs of different plant species.

Ten subfamilies (CI, CII, CIII, CV, CVI, CVII, MII, CP, ER, and Po) of the PpHSP20 family contained 18, 2, 2, 10, 5, 1, 1, 2, 1, and 2 proteins, respectively. Eighteen out of the 44 *PpHSP20* genes (40.91%) were classified into the CI subfamily. Subfamily CI was the largest subfamily in the PpHSP20 family of the peach genome. This is consistent with previous findings indicating that the CI gene subfamily is generally the largest HSP20 subfamily in plants [11]. This feature has also been reported in *Arabidopsis*, potato, pepper, grape, and tomato. These results indicated that subfamily CIs are evolutionarily conserved. Additionally, subfamilies CI and CII appeared on the first branch of the phylogenetic tree (Fig. 1) in this study. These results indicated that subfamilies CI and CII are ancient and evolutionarily conserved. As indicated by previous findings in a bryophyte, the presence of members of the cytosolic I and II HSP20 families indicates that these gene families are ancient and evolved at least 450 million years ago [8].

Gene structure plays a key role in the evolution of a given multiple-gene family among different plant species. In this study, 16 out of 44 *PpHSP20* genes had one exon and no introns (Fig. 4). The percentage (36.36%) of intronless *PpHSP20* genes was similar to that found in potato (39.58%) [16], pepper (45.71%) [22], and tomato (30.95%) [7]. Furthermore, the *PpHSP20* genes of the CII subfamily as well as most *PpHSP20* genes of the CI and ER subfamilies had one exon and no introns (Fig. 4). The members of the CV, MII, and CP subfamilies and most members of the CVI subfamily had two exons and only one intron. To some degree, these results were consistent with those observed in potato, pepper and tomato. The expression levels of genes with few or no introns were higher than those of other genes in plants. This may be due to the need to respond to various stresses in a timely manner, as the rapid activation of genes requires a compact structure with fewer introns [29, 30].

It is already well established that, at least in eukaryotes, the expansion of gene families and genome evolution mechanisms mainly depend on gene duplication events [31, 32]. In this study, approximately 15 of the *PpHSP20* genes were located in the two terminal regions or at one terminus of each chromosome (Fig. 5). Some *HSP20* genes preferentially localize to the terminal regions, which is also observed in species such as grape and potato [13, 16]. A total of 15 tandem *PpHSP20* genes were identified in peach, which belonged to five tandemly duplicated gene groups (Fig. 5). These results suggested conservation between these tandemly arrayed copies. This may be due to horizontal gene transfer or unequal crossing over [32]. Tandem duplication might facilitate peach fruit adaptation to various environmental changes under a dynamic and reversible regulatory mechanism [32]. The tandem duplication of these genes might act as an evolutionary force driving the origination and maintenance of these clusters.

### Expression profiling of PpHSP20 family genes

Two members of the subfamily CVI, *Prupe.5G186700.1* or *Prupe.7G053900.1*, showed the same expression trend in each cultivar (red flesh, yellow flesh, and white flesh), respectively (Fig. 7). This implied that each gene might play the same role in a certain trait or characteristic in all three cultivars. This could imply that both *Prupe.5G186700.1* and *Prupe.7G053900.1* do not have functions in the coloration of red flesh.

Two members of subfamily CP, *Prupe.1G237800.1* and *Prupe.2G317700.1*, showed a high expression trend in the red-fleshed cultivar and a low expression trend in the yellow- and white-fleshed cultivars (Fig. 7). This could imply that both *Prupe.1G237800.1* and *Prupe.2G317700.1* might have functions in the red coloration of fruit flesh. This also indicated that there is a close relationship between gene expression and subfamily classification and between gene expression and amino acid sequence homology.

No expression of *Prupe.1G407200.1* or *Prupe.8G046200.1* was detected in any of the three peach cultivars at any developmental stage (Fig. 7). This finding implied that these genes may not function in the development of peach flesh. However, they may function in other tissues or in biotic and abiotic stress responses. For example, one HSP20 of sugarcane is only expressed during stress and is involved in the regulation of transcription and posttranslational modifications [5].

Overall, the number of *PpHSP20* genes with higher expression levels in red-fleshed ‘BJ’ was greater than that in yellow- or white-fleshed cultivars. These results indicated that some *PpHSP20* genes might contribute to red color formation in peach flesh. This was somewhat similar to the results obtained in tomato, in which seven *SlHSP20* genes showed differential expression between mature green and red ripe fruits. Additionally, it has been reported that *SlHSP21* can promote color change during fruit ripening and play a key role in the transformation of chloroplasts to pigmented mother cells during fruit ripening [33, 34]. However, some *PpHSP20* genes were associated with certain developmental stages. These results were consistent with previous reports in other plant species, such as grape, in which it has also been implied that *VvHSP20* genes are involved in fruit development [13]. Overall, the expression pattern of *PpHSP20* genes indicated that *PpHSP20* genes may have diverse functions and play critical roles in the development of red- flesh, which still deserves further exploration.

Our previous studies demonstrated that the presence of red flesh in ‘BJ’, due to anthocyanins, is the result of a large accumulation of cyanidin-3-O-glucoside, with slight cyanidin-3-O-rutoside [35]. Anthocyanin was not detected until approximately 70 DAFB in ‘BJ’ and then increased continuously from approximately 70 to 87 DAFB before reaching its maximum [35]. Thus, S3 (70 DAFB) is a very important transformation period from white color to gradual anthocyanin accumulation (red color) in ‘BJ’. In this study, some *PpHSP20* genes only showed high expression at the S3 of red-fleshed ‘BJ’ and low expression at the S3 of white- and yellow-fleshed cultivars, namely just at the beginning period of anthocyanin accumulation (Fig. 7). These results indicated that some *PpHSP20* genes might play vital roles in the initiation of anthocyanin accumulation in the red-fleshed ‘BJ’. After fulfilling this mission at S3, they look like fade away at S4-1 and S4-2 (Fig. 7). Some unknown mechanisms of initiating anthocyanin accumulation in the red flesh of ‘BJ’ still need to be explored. In the next stage, we will focus on whether and how some *PpHSP20* genes (*Prupe.2G243400.1*, *Prupe.6G066200.1*, *Prupe.1G586200.1*, *Prupe.1G237800.1*, *Prupe.2G317700.1*, and *Prupe.5G005900.1*) are involved in anthocyanin biosynthesis and red flesh development through more in-depth experiments.

### Subcellular location of PpHSP20 family proteins

To check whether the subfamily classification based on subcellular localization complied with the real subcellular location, the subcellular localizations of 19 PpHSP20 proteins selected from subfamilies CI, CII, CIII, CV, CVI, MII, Po, and CP were analyzed in this study. As a result, the subcellular localizations of most of the 16 identified PpHSP20 proteins were in accord with the subfamily classifications of PpHSP20s and the principles of CI, CII, CIII, CV, CVI, CP, and MII subfamily naming originating from *Arabidopsis*. The remaining three (Prupe.1G586200.1, Prupe.8G046200.1, Prupe.8G000400.1) of the 19 PpHSP20s were exceptions to these findings.

Prupe.3G034800.1, which was classified into subfamily CIII according to phylogenetic relationships and homologous genes in grape and *Arabidopsis* (Fig. 1), was targeted in both the nucleus and endoplasmic reticulum in this study (Fig. S1). This result was consistent with previous reports indicating that the localization of CIII HSP20s is primarily nuclear [33]. On the other hand, this result verified previous reports about CIII HSP20 proteins indicating that nuclear localization is conserved in CIII HSP20s [33]. However, for dual targeting to both the endoplasmic reticulum and nucleus of Prupe.3G034800.1, to date, this is the first report for HSP20s in plants to our knowledge. Dual targeting of proteins to two organelles is increasingly being reported with the development of techniques for the assessment of subcellular localization. For instance, dual targeting to both mitochondria and the nucleus was observed for AT2G41380 in previous literature [36]. AT5G51440.1 (sHSP23.5) and AT4G25200.1 (sHSP23.6) were found to be dual targeted to both mitochondria and chloroplasts [36], although they had previously been assumed to be only mitochondrial [37].

In the 1980s and 1990s, subcellular localization was mainly analyzed by cell fractionation and a linear sucrose gradient [38, 39]. With the development of biological techniques, reporter genes (such as green fluorescent protein, GFP) have been used to detect the subcellular localization of proteins [11, 23, 40]. With this technique, subcellular localization analysis of OsSHSP1 showed that it targeted the cytosol, whereas OsSHSP2 targeted the cytosol and nucleus [10]. Five new subfamilies (CIV, CV, CVI, CVII, MII) of *Arabidopsis* HSP20s were also verified through analysis of subcellular localization by transient transformation of vectors with GFP reporter genes [11]. Using this method, the subcellular localization of a given protein is clearer and more intuitive than in the old method.

The subcellular localizations of Prupe.1G237800.1 in subfamily CP and Prupe.3G085200.1 in subfamily MII were almost completely in accord with the subfamily classification and phylogenetic relationship of grape and *Arabidopsis* (Fig. 1 and Fig. S1). Chloroplast and mitochondrial HSP20s are considered to play an important role in heat tolerance [39, 41]. The discovery of Prupe.1G237800.1 in chloroplasts and Prupe.3G085200.1 in mitochondria in this study is a prerequisite to further study their function in flesh development. Six out of the eight analyzed PpHSP20 proteins of subfamily CI had the same subcellular localizations as the previous report in *Arabidopsis* to a certain degree [11], which were located in the cytoplasmic and/or nuclear compartment. Meanwhile, these six proteins were also found to be located in the plasma membrane, which was not reported for the corresponding homologous proteins in *Arabidopsis*. Thus, it can be indicated that to a certain degree, the subfamily classification and subfamily name of these six PpHSP20 proteins in subfamily CI comply with the real subcellular location.

In this study, there were also some exceptions, which were not in accordance with the subfamily classification and phylogenetic relationship of grape or *Arabidopsis* (Fig. 1 and Fig. S1). According to the phylogenetic relationship and homologous proteins (Fig. 1), Prupe.1G586200.1 and Prupe.8G046200.1 clustered together with AT4G10250.1 (ER protein), VIT_218s0001g01570.1 (HSP20-45, Px/Po protein), and VIT_218s0089g01270.1 (HSP20-42, ER protein) (Fig. 1). Meanwhile, Prupe.8G000400.1 was closely next to VIT_218s0089g01270.1 (HSP20-42, ER protein) and slightly far from VIT_218s0001g01570.1 (HSP20-45, Px/Po protein) (Fig. 1). Additionally, Prupe.1G586200.1, Prupe.8G046200.1, and Prupe.8G000400.1, together with the above homologous proteins, were on the same branch. Therefore, we initially classified Prupe.1G586200.1, Prupe.8G046200.1, and Prupe.8G000400.1 into the ER subfamily. Thereafter, the subcellular localization results (Fig. S1) indicated that Prupe.1G586200.1 was located in the plasma membrane and cytoplasm, and Prupe.8G046200.1 was located in the plasma membrane, nucleus, and cytoplasm (Fig. S1). The subcellular localization of Prupe.8G000400.1 was in the plasma membrane, cytoplasm, and peroxisome (Fig. S1). Thus, the real subcellular localization of Prupe.1G586200.1, Prupe.8G046200.1, and Prupe.8G000400.1 was not in accordance with the subfamily classification of ER and phylogenetic relationship. Finally, according to the real location of experimental evidence, Prupe.1G586200.1 and Prupe.8G046200.1 were classified into neighboring subfamily CV, while Prupe.8G000400.1 was classified into subfamily Po (Fig. S1). The same cases were also found in the literature [37, 40, 41]. For instance, AtHSPl5.7 (AT5G37670.1) was previously reported to belong to cytosolic class I [37]. Recently, experimental evidence using onion epidermal cells demonstrated that it localized in the peroxisome and belongs to the Po/Px subfamily [40].

## Conclusions

In total, 44 PpHSP20 members yielded in this study were divided into 10 subfamilies, CI, CII, CIII, CV, CVI, CVII, MII, ER, CP, and Po, containing 18, 2, 2, 10, 5, 1, 1, 1, 2, and 2 proteins, respectively. To a certain degree, the subcellular localization of 19 PpHSP20 proteins verified the correction of subfamily classification of PpHSP20s and provided the necessary foundation for further functional verification. Overall, the number of *PpHSP20* genes with the higher expression levels in red flesh were greater than that in yellow or white flesh among the three cultivars. Compared with S2, S4-1, and S4-2 stages, the expression profiling of most *PpHSP20* genes in red-fleshed ‘BJ’ was relatively higher overall at the S3 stage, which is a very important period of transitioning from a white color to a gradual anthocyanin accumulation (red color) in the flesh of this cultivar. We proposed that some *PpHSP20* genes might play vital roles in the initiation of anthocyanin accumulation (red color) in the red-fleshed ‘BJ’. This report lays a foundation for future analyses of the unknown function of PpHSP20 family genes in red-fleshed peach fruits and advances the knowledge of plant HSP20 family genes.

## Materials and methods

### Plant materials

For expression profiling, we used transcriptome data submitted by a team member [42]. The selected experimental materials were as follows: fruits from one-year-old fruiting shoots from the outer southern canopies of ‘XiaHui5’ (‘XH5’, white flesh), ‘RuiGuang18’ (‘RG18’, yellow flesh), and ‘BeiJingYiXianHong’ (‘BJ’, red flesh) peach trees grown under standard field conditions at the National Peach Germplasm Repository in Nanjing, China. According to the growth and development curve of peach fruits, four developmental stages (pit hardening (S2), expansion stage (S3), premature stage (S4-1), and mature stage (S4-2)) were included in transcriptome sequencing. Among the three cultivars, fruits from ‘BJ’ were selected at 50, 70, 80, and 90 days after full bloom (DAFB); fruits from ‘XH5’ at 60, 80, 90, and 96 DAFB; and fruits from ‘RG18’ at 80, 100, 110, and 120 DAFB [42]. All of the fruit samples were peeled, and the flesh was immediately frozen in liquid nitrogen, followed by storage at -80 °C.

‘BJ’ fruit without the peel collected at 90 DAFB was used as the material for the cloning and subcellular localization of 19 *PpHSP20* genes. All plant experiments were performed in accordance with national/institutional guidelines and regulations.

### Identification of PpHSP20 family members

The genome sequence, CDS, protein sequence, and transcript sequence of peach ‘Lovel’ version 2.0 were downloaded from the GDR database, respectively. The accession number of the HSP20 domain (PF00011) specific to the plant HSP20 family was obtained from the Pfam database [43]. Then, PpHSP20 family members were predicted using HMMER [44] software. A conserved domain search (CD search) [45] and InterProScan [46] tools were used to further confirm the integrity of the HSP20 domain (also called the ACD domain) for each predicted HSP20 family member in peach. The amino acid sequence of each PpHSP20 protein was uploaded to the BLAST box in the TAIR database to obtain the corresponding most homologous HSP20 genes in *A. thaliana*.

### Phylogenetic analysis of HSP20 proteins

Nineteen and 48 HSP20 family protein sequences in *A. thaliana* and grape were obtained from The Arabidopsis Information Resource (TAIR10) and Ensembl Plants databases, respectively, based on previous work [11, 13]. Thirteen and 13 HSP20 family protein sequences of *M. domestica* and *M. sieversii* were downloaded from the NCBI and GDR databases, respectively. The ID of each HSP20 protein from grape, *A. thaliana*,* M. domestica*, and *M. sieversii* is listed in Table S1.

To make subfamily classification of the PpHSP20 proteins and demonstrate the phylogenetic relationships among the HSP20 members in peach, grape, *A. thaliana*, *M*. *domestica*, and* M*. *sieversii*, a circular phylogenetic tree composed of HSP20 protein sequences for peach, grape, *A. thaliana*, *M. domestica*, *and M. sieversii* was generated using MEGA 4.1[47]. The  parameter settings were neighbor-joining, p-distance, and 1000 replicates (bootstrap). Based on the phylogenetic tree (Fig. 1), subcellular location and subfamily naming method of references in *Arabidopsis*, apple, grape, and other plant species, the PpHSP20 proteins identified in this study were classified into corresponding subfamilies.

### Analysis of the motif, exon/intron, and physiochemical characteristics of PpHSP20 members

The conserved motif location, exon/intron location, and physiochemical characteristics of PpHSP20 were analyzed using the online tools MEME (V4.12.0) [48], GSDS [49], and ExPASy [50], respectively, as described in a previous study [24]. The IDs of PpHSP20 in the generated figures were arranged in order of those in the phylogenetic tree.

The length of four kinds of sequences (genomic sequence, coding sequence, transcript sequence, protein sequence), position on the chromosome, and ID for v1.0 and v2.0 of each PpHSP20 were obtained from the phytozome and GDR databases.

The distribution of *PpHSP20* genes on the eight chromosomes (Table S3) was visualized using the software MapDrawV2.1 [51]. The collinearity map was generated with the circlize tool in the R package. The information for length of each chromosome was obtained from the GDR database.

### Cloning and subcellular localization analysis of 19 PpHSP20s

Based on the phylogenetic tree and subfamily classification, we selected 19 proteins (Prupe.1G407300.1, Prupe.2G243800.1, Prupe.2G243400.1, Prupe.6G066300.1, Prupe.6G066500.1, Prupe.6G065900.1, Prupe.6G066100.1, Prupe.4G125800.1, Prupe.1G407200.1, Prupe.3G017400.1, Prupe.1G586200.1, Prupe.8G046200.1, Prupe.8G000400.1, Prupe.5G071200.1, Prupe.1G237800.1, Prupe.3G085200.1, Prupe.3G034800.1, Prupe.6G008800.1, Prupe.7G053900.1) from subfamilies CI, CII, CIII, CV, CVI, MII, CP, and Po for further gene cloning and subcellular localization analysis.

Total RNA extraction from the red flesh of the ‘BJ’ cultivar and reverse transcription of RNA were conducted using a Plant RNA Kit (TaKaRa Biotechnology Co. Ltd., Dalian, China) and the RT Reagent Kit (TaKaRa Biotechnology Co. Ltd., Dalian, China), respectively. All the cDNA samples were diluted to 100 ng μl^−1^ and were used as templates for ORF cloning of the above 19 genes. The primers used are listed in Table S2. The construction of each vector with the GFP reporter gene and transient transformation for each *PpHSP20* was performed as described in our previous report [25]. Transiently expressed PpHSP20-GFP in leaf epidermal cells of *N. benthamiana* was observed using an Ultra VIEW VoX confocal laser scanning microscope (PerkinElmer Instruments (Shanghai) Co. Ltd., Shanghai, China).

### Expression profiling of *PpHSP20* family genes

Transcriptomic sequencing of three cultivars was performed by another team member [42]. The estimation of gene expression levels was performed based on the expected number of fragments per kilobase of transcript sequence per million base pairs sequenced (FPKM).

To gain insight into the expression profiles of the 44 *PpHSP20* family genes from young green fruits to mature fruits, the average FPKM values of three biological repetitions of each developmental stage in each cultivar for each *PpHSP20* gene were extracted from the transcriptomic data [42]. The expression profiles of the 44 *PpHSP20* genes are displayed in a heatmap generated by the Pheatmap (R package) tool.

### Supplementary Information

**Additional file 1:** **Table S1.** Summary of each HSP20 protein from four plant species used for construction of the phylogenetic tree.

**Additional file 2:** **Table S2. **Sequences of primers designed for ORF cloning of 19 PpHSP20 genes from red-fleshed ‘BJ’ fruit.

**Additional file 3:** **Table S3.** Summary of PpHSP20 family genes in peach.

**Additional file 4:** **Table S4.** Summary of physicochemical properties of PpHSP20 proteins.

**Additional file 5:** **Table S5.** Summary of collinearity pairs of HSP20 family genes in peach and *Arabidopsis thaliana*.

**Additional file 6:** **Figure S1. **Subcellular localization of 19 PpHSP20 proteins.

## Data Availability

All the data generated or analyzed during this study are included in this published article and its supplementary information files. The peach sequences in this article can be found in GDR (https://www.rosaceae.org/). The *Arabidopsis thaliana* sequences in this article were downloaded from TAIR (https://www.arabidopsis.org/). The expression profiling data presented in this study are deposited in the NCBI SRA repository under accession number PRJNA850935 (https://submit.ncbi.nlm.nih.gov/subs/sra/SUB11621949/overview). ‘XH5’ was bred and identified first by researcher Mingliang Yu in 2003 at the Institute of Pomology, Jiangsu Academy of Agricultural Sciences, Nanjing City, Jiangsu Province, China. ‘XH5’ was assigned voucher number B032. ‘RG18’ was bred and identified first by researchers Quan Jiang and JiYing Guo in 1999 at the Institute of Forestry and Pomology, Beijing Academy of Agriculture and Forestry Sciences, Beijing City, China. ‘RG18’ was introduced into the National Peach Germplasm Repository in Nanjing by researcher Mingliang Yu in 1999 and was assigned voucher number N017. ‘BJ’ was identified first by researcher Quan Jiang in 1990 at the Institute of Forestry and Pomology, Beijing Academy of Agriculture and Forestry Sciences, Beijing City, China. ‘BJ’ was introduced into the National Peach Germplasm Repository in Nanjing by researcher Mingliang Yu in 1999 and was assigned voucher number Y033. Voucher specimens of all plants were deposited in the public herbarium of the National Peach Germplasm Repository in Nanjing, which is located in the Jiangsu Academy of Agricultural Sciences, Nanjing City, Jiangsu Province, China.

## Abbreviations

ACD: α-Crystalline domain
CDSs: Coding sequences
C: Cytosolic/nucleus
CP: Chloroplasts
DAFB: Days after full bloom
GDR: Genome database for Rosaceae
ER: Endoplasmic reticulum
GSDS: Gene structure display server
HMM: Hidden Markov model
HSPs: Heat shock proteins
kDa: Kilodaltons
LMW proteins: Low-molecular-weight proteins
MT or M: Mitochondria
MEME: Multiple Em for Motif Elicitation
ORF: Open reading frame
P: Plastid
PM: Plasma membrane
Px or Po: Peroxisomes
sHSP: Small heat shock protein
TAIR: The Arabidopsis Information Resource
XH5: XiaHui5
BJ: BeiJingYiXianHong
RG18: RuiGuang18
