# Supplementary figures and images for: Integrated analysis of HSP20 genes in the developing flesh of peach: identification, expression profiling, and subcellular localization
Source: BMC Plant Biol. 2023 Dec 21;23:663. doi: 10.1186/s12870-023-04621-0 (PMC10740231; doi:10.1186/s12870-023-04621-0)

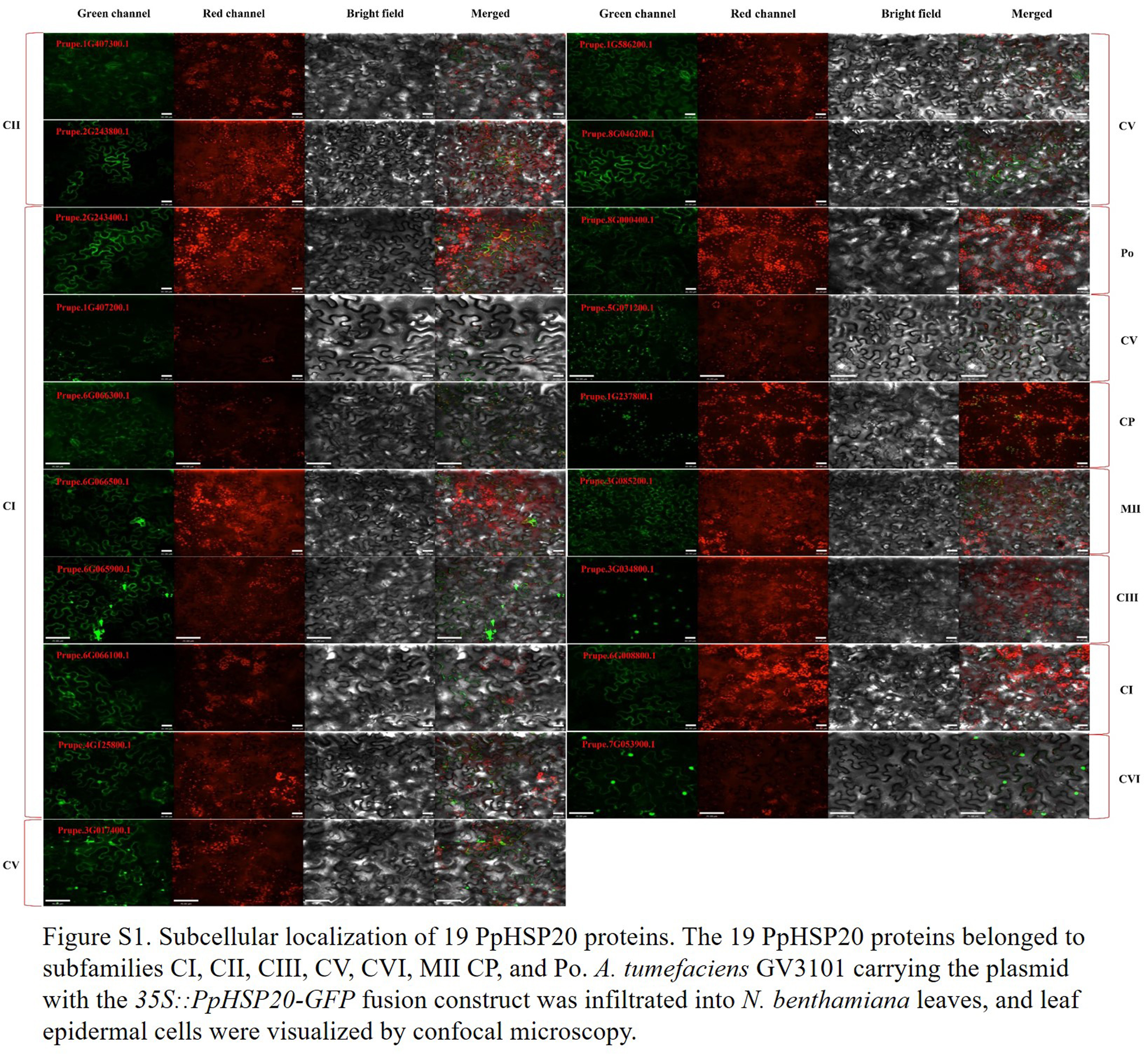

Supplement: Supplementary file 6 — Additional file 6: Figure S1. Subcellular localization of 19 PpHSP20 proteins. [file 12870_2023_4621_MOESM6_ESM.jpg]
